# Global Longitudinal Strain May Be the One that Appropriately Identifies Candidates of ICD Implantation

**DOI:** 10.1155/2024/2214072

**Published:** 2024-01-16

**Authors:** Mohammad Hossein Nikoo, Mohammad Zarrabi, Alireza Moaref, Iman Razeghian-Jahromi

**Affiliations:** ^1^Cardiovascular Research Center, Shiraz University of Medical Sciences, Shiraz, Iran; ^2^Department of Cardiovascular Medicine, School of Medicine, Shiraz University of Medical Sciences, Shiraz, Iran; ^3^Non-Communicable Diseases Research Center, Shiraz University of Medical Sciences, Shiraz, Iran; ^4^Student Research Committee, Shiraz University of Medical Sciences, Shiraz, Iran

## Abstract

Hypertrophic cardiomyopathy (HCM) significantly contributes to an elevated risk of sudden cardiac death. Primary prevention is implemented by using an implantable cardioverter defibrillator (ICD). However, all of the HCM patients do not really need ICD therapy. Providing a superior index for ICD indication compared with the current indices like ejection fraction is essential to differentiate high-risk patients efficiently. The present study assessed the potential of global longitudinal strain (GLS) for the differentiation of HCM patients based on their need for ICD shocks. Patients with HCM were considered in four defined centers between March and June 2021. Those with previous ICD implantation or current candidates for ICD therapy were included in the study. Participants were subjected to speckle-tracking echocardiography, and GLS as well as some other echocardiographic parameters were recorded. Afterwards, data from implanted ICDs were extracted. Patients who received ICD shocks (appropriate) due to ventricular tachycardia (VT)/ventricular fibrillation (VF) were categorized in group A. The remaining patients were constituted group B who received inappropriate shocks, i.e., other than VT/VF. Overall, 34 patients were found eligible to participate with a mean age of 62 ± 16.1 years including 64.7% of males. Among a variety of echocardiographic parameters, GLS was the sole one that was significantly higher in group A compared with that in group B. Our findings revealed that only GLS could predict fatal arrhythmias. To substantiate, the odds of VT were raised by 43% with a single increase in GLS unit. GLS showed the highest accuracy for ICD indication among HCM patients and, therefore, could be a solid and early criterion to predict the incidence of life-threatening arrhythmias. In this regard, identifying appropriate HCM patients with respect to their need for ICD therapy is feasible.

## 1. Introduction

Hypertrophic cardiomyopathy (HCM), the most common hereditary cardiomyopathy, is linked to an elevated risk of cardiovascular morbidity and death [1]. In particular, patients with HCM have a higher risk of sudden cardiac death (SCD) and death owing to heart failure [2, 3]. A wide range of phenotypes with variable prognoses, from life-long asymptomatic ones to SCD at young ages, make risk stratification of HCM a sophisticated task. The current strategy for such patients is mostly focused on SCD prevention [4, 5]. The survival rate of patients with HCM-originated heart failure is significantly increased by using an implantable cardioverter defibrillator (ICD) as a primary prevention method [6–9]. ICD therapy, however, comes along with financial considerations and increased hospitalizations [10]. Moreover, ICD may be implanted in patients who never receive therapeutic shocks thereafter [6], or there are instances of patients with low ejection fraction and concurrent low risk of SCD [7, 11]. These pieces of evidence show low sensitivity and specificity of the current measures [12]. As a result, researchers have concentrated their efforts on discovering new potent prognosticators in order to improve the efficiency of ICD therapy in HCM patients [13–18].

Speckle-tracking echocardiography (STE) distinctively measures the length of myocardial segments by tracking the displacement of myocardial speckles [19]. The most usual unit in STE, strain, represents alterations in the myocardial fiber length at end systole compared to end diastole. Global longitudinal strain (GLS), derived from longitudinal, radial, and circumferential strains, detects and quantifies fine LV disturbances that reflect systolic function [20]. This outstanding technique is non-Doppler-oriented and possesses an angle-independent nature. Also, it is less influenced by ventricular loading, compliance of myocardium, and afterload properties [21].

To investigate the potential of GLS as an index for predicting fatal arrhythmias, HCM patients were assessed based on the shocks received by the implanted ICDs, and then, the appropriateness of the shocks was evaluated. Also, a cut-off value is suggested for GLS which could be further useful for ICD indication in HCM patients.

## 2. Methods

This study complies with the Declaration of Helsinki and was approved by the regional ethics committee. All the patients signed informed written consent before participation. HCM was defined as a maximal LV hypertrophy (LVH) of more than 15 mm in the absence of any other cardiac or systemic conditions that could generate a similar degree of LVH [5, 22]. Such patients were recruited from four defined centers between March and June 2021. Those with previous ICD implantation or current candidates for ICD therapy were included. Patients who experienced the following conditions in the past or during the follow-up period were excluded from the study: septal myectomy, septal alcohol ablation, coronary artery disease (in terms of positive exercise tolerance test, stenosis of more than 50% according to coronary angiography, revascularization including percutaneous coronary intervention/coronary artery bypass grafting, and documented myocardial infarction), and myocardial hypertrophy due to specific reasons such as hypertension, valvular heart disease, storage disease, or pulmonary disease. Also, participants were not using certain medications, including bisoprolol, metoprolol, propranolol, amiodarone, disopyramide, diuretics, and calcium channel blockers.

An expert with a subspecialty in echocardiography, who was blinded to the experiment, performed STE (GE Vivid E9, GE Healthcare, Horten, Norway). Image analysis was carried out on recorded data by EchoPAC, GE Medical Systems, Horten, Norway. Echocardiographic parameters including GLS, left ventricular end-systolic diameter and left ventricular end-diastolic diameter (LVESD and LVEDD), interventricular septal thickness (IVST), posterior wall thickness (PWT), left ventricular ejection fraction (LVEF), maximum wall thickness (MVT), and left atrium (LA) diameter were measured. The longitudinal strain was measured based on the mean of strains in four, two, and three apical chamber views in the frame rate of 50−60s. GLS was calculated as the mean of peak longitudinal strain in 16 segments in the left ventricular wall. According to the American College of Cardiology/American Heart Association guideline, the parasternal long axis was used in M-mode acquisition to calculate left ventricular wall thickness in 2D imaging at the end of systole and diastole. Likewise, the diameter of the left and right atria was measured [22]. EF was calculated based on modified Simpson's method (biplane method of disks). PWT [23] and IVST [24] were measured, and each of them that was higher was assumed as MWT [22].

About 72 hours after echocardiography, interrogation of implanted ICDs was carried out with a compatible analyzer. Medtronic ICDs were used in 30 patients, and the remaining four were implanted with St. Jude devices. The events that led to therapy were sought to see whether the shocks were appropriate or not. Patients were classified into two groups: group A are those who received appropriate shocks due to sustained VT/VF, and group B are those who received inappropriate shocks including atrial tachycardia (AT), atrial fibrillation (AF), and other reasons or did not receive any shock at all. All the participants were followed up for at least one periodic visit. Furthermore, some participants were subjected to gadolinium-enhanced cardiovascular magnetic resonance (CMR) by a 1.5T Ingenia machine (Philips Co.). Scar burden, EF value, the status of the cardiac valves, and left ventricular outflow tract (LVOT) were assessed in these patients.

### 2.1. Statistical Analyses

SPSS version 22 (IBM corporation, Armonk, NY, USA) was used for statistical analyses. The normal distribution of the data was measured by the Shapiro–Wilk test. Continuous variables are shown as mean ± standard deviation. Independent sample t-test, Mann–Whitney *U* test, and ANOVA were used to compare differences between groups, followed by the LSD post hoc test. The specificity and sensitivity of variables for the prediction of VT/VF occurrence were analyzed by the receiver operating characteristic (ROC) curve. Correlation coefficient and binominal logistic regression were used for assessing the correlation between GLS and the incidence of VT/VF. A *P* value of less than 0.05 was considered as statistical significance. Youden's index was used in ROC curve analysis to determine the optimal cut-off point for GLS in predicting the appropriate shocks.

## 3. Results

Out of 45 patients who were referred to the four centers during the time frame, 34 were included in the study with a mean age of 62 ± 16.1 years and 64.7% of males. Men were older (64.7 ± 13.8) than women (47.4 ± 15.8). Different medications that were used by the patients at the time of enrollment were provided in Table 1. All the patients were receiving optimal medical therapy according to the current guidelines, and none of them were taking amiodarone or other antiarrhythmic drugs.

Table 2 demonstrated the echocardiographic parameters of the participants retrieved by the STE.

Out of the 34 patients enrolled, 19 had previous implantation of ICD and 15 were candidates for ICD therapy. The mean time from the implantation to the enrollment of the patients who were already on ICD therapy was 18.4 ± 9.6 months with a range of 6 to 36 months. It should be noted that candidates for ICD therapy have been followed up for a median of 36 months (interquartile range of 24–48 months), which is comparable to that of the patients with previous ICD implantation.

Only those shocks for ventricular tachycardia (VT) or ventricular fibrillation (VF) were considered as the appropriate ones (group A). Echocardiographic parameters were compared between two groups (Table 3). As shown, GLS was the only parameter that was significantly different in group A compared with the peers in group B.

**Figure 1:**
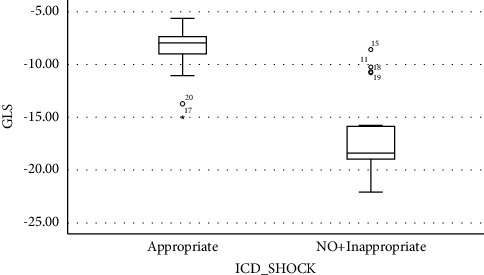
Box plot demonstrating groups A and B based on the GLS values and the type of shocks received by ICDs.

Figure 1 demonstrates a box plot based on the GLS values (%) and the type of shock received by the patients. Patients in group A (who received appropriate shocks) had higher GLS values on average. There was no significant association between GLS and scar burden possibly because of the small sample population (data was not shown).

In order to find which parameter was a better predictor for the incidence of arrhythmia, binominal logistic regression was used.  Table 4 depicts the potential of different variables for predicting VT incidence. Findings revealed that GLS was the only parameter that significantly predicted fatal arrhythmias (*P*=0.032). With a single increase in GLS unit (one unit decrease in the absolute value), the odds of VT incidence were raised by 43%. Results show that the logistic regression model is statistically significant (*P*=0.013), and 67.8% of the variances of the appropriate shocks (Nagelkerke *R*^2^=67.8%) were explained by this model. Indeed, this model has classified 78.3% of the total shocks into appropriate groups.

**Figure 2:**
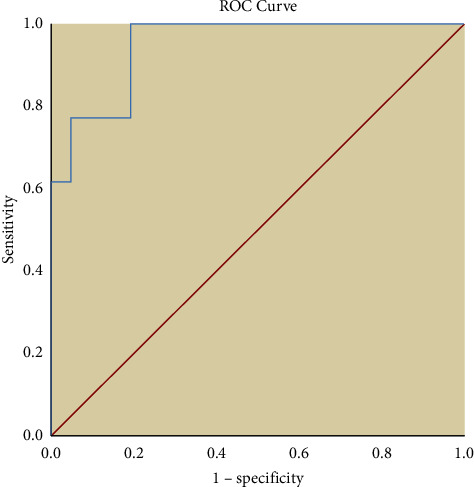
ROC curve to reveal sensitivity and specificity of GLS in predicting the need for ICD therapy.

Based on the findings from the ROC curve, GLS accurately defines those patients needing ICD to prevent VT incidence from those patients not benefiting from it (Figure 2) (AUC = 0.949, *P* < 0.001). It was revealed that GLS of −14.4% had the sensitivity and specificity of 92.3% and 81% for such prediction.

## 4. Discussion

In the present study, the incidence of fatal arrhythmias, both ventricular and supraventricular, was retrieved from implanted ICDs in HCM patients during a period of six months. Sustained VT and VF were sought and considered as appropriate shocks, and other types of shocks were regarded as inappropriate ones. Having all the patients subjected to STE, GLS was the only significant difference between the two groups. This finding is of paramount importance because other parameters previously considered important predictors of arrhythmia, like that of diastolic (LA diameter and left ventricular filling pressure) and systolic ones (left ventricular wall thickness and EF), were not significantly different between the two groups. It emphasizes the potential and value of STE-derived GLS for predicting arrhythmias, and hence, as a criterion for ICD therapy in HCM patients as well as patients' prognosis. AUC of 0.949 supports the worth of GLS for the prediction of arrhythmias. Our findings also revealed that a cut-off value of −15.45% for GLS is useful for screening HCM patients, and that of below 8.35% is an independent and definite indicator for ICD implantation in HCM patients (with a specificity of 100%). Moreover, it was shown that GLS is not influenced by age and gender in our studied population, representing the potential applicability of this index irrespective of demographic variables in HCM patients.

HCM is a hereditary autosomal dominant disease characterized by the increased risk of LVH incidence, cardiac fiber disarray, and interstitial fibrosis [25, 26] that ultimately leads to impairment in cardiac systolic function. Cardiac output, however, remains within the normal limits in some instances. Accurate risk stratification is a clinical problem in patients with HCM. SCD prevention, in which its risk is currently justified based on certain markers such as family history, unexplained syncope, nonsustained VT, LV thickness, and LVOT gradient, is the major target in these patients [27]. Also, the overall prognosis of HCM patients is in relation to N-terminal pro-B-type natriuretic peptide, the existence of atrial fibrillation, the New York Heart Association class, and the result of the functional exercise capacity test [15–17]. Furthermore, myocardial fibrosis, which is determined by cardiovascular magnetic imaging with late gadolinium enhancement, is a key risk factor that has been attributed to both SCD and severe cardiovascular events in individuals with HCM [13, 14, 18, 26]. Besides multiple limitations that restrain these measures, simple and readily available markers that represent anatomical problems such as cardiac fibrosis, as well as systolic and diastolic dysfunctions, would be the ideal prognosticators in clinical settings.

STE is a very sensitive technique to probe strains of cardiac muscles in order to measure myocardial alterations directly, either segmental or global [26]. GLS is a parameter that shows longitudinal contraction of the myocardium, and its accuracy has been confirmed using the magnetic resonance imaging approach [28]. The importance of longitudinal strain in predicting arrhythmic episodes was emphasized in patients with myocardial infarction [29, 30]. Low GLS is associated with sustained or nonsustained VT incidence. Indeed, STE-derived GLS helps detect cardiac abnormalities earlier before cardiac output becomes impaired [31, 32]. This parameter has been found to detect mild myocardial dysfunction in individuals with HCM in several studies, which are likely the reflection of myocardial fiber disarray, fibrosis, and microvascular dysfunction [32] as GLS and fibrosis were shown to be in close association with HCM patients who underwent septal myectomy. Furthermore, it was declared that GLS was even a better predictor of arrhythmias compared with cardiovascular magnetic imaging with late gadolinium enhancement [33]. GLS measurement has some advantages in terms of operator independence, reproducibility (particularly in comparison with EF), and integration with regular echocardiography [28].

There is no strong consensus regarding the criteria for ICD indication in HCM patients between the American Heart Association and the European Society of Cardiology. Current LVEF-based primary prevention has some limitations concerning low reproducibility and feasibility [34], poor representation of myocardial contractility, being normal due to compensatory over-contraction in some cases of cardiac diseases [35], association with geometry and operator's experience, and low sensitivity in detecting the risk of VT/VF [9, 36]. Moreover, it was shown that LVEF of <35% has appeared only in 20% of those who died because of SCD [37]. On the contrary, there are patients with LVEF of <35% and concurrent low risk of SCD occurrence [11]. So, it is necessary to define an index to differentiate HCM patients more appropriately in terms of their need for ICD implantation. In one study, GLS, besides wall thickness and fibrosis, was associated with ventricular arrhythmias in 150 HCM patients [38].

GLS was nominated as the best parameter to create a mortality risk prediction model for individuals with acute heart failure syndrome in a major study by Hwang and colleagues on 4312 patients [39]. In a large cohort of 427 HCM patients, GLS was shown to be independently associated with all-cause mortality, heart transplantation, aborted SCD, and appropriate ICD therapy [1]. It was reported that GLS could be an indirect indicator of fibrosis and cardiac fiber disarray. In this regard, all the patients who received appropriate shock had GLS of more than −14%. Sensitivity and positive predictive values were reported to be 100% and 24%, respectively [40]. However, in the present study, sensitivity and positive predictive values were 72.8% and 80%, respectively.

Three-dimensional speckle-tracking imaging demonstrated that GLS is a potent tool to efficiently predict cardiac events such as heart failure, syncope, and sudden cardiac death. Patients with three-dimensional GLS of ≤13.67% experienced more SCD events than those of >13.67% [41]. GLS of higher than −16% was shown to predict heart failure hospitalizations, sustained ventricular arrhythmias, and all-cause mortality [42]. Perry and colleagues evaluated 1014 patients and identified that the best cut-off value for GLS is −15% using ROC analysis. Furthermore, GLS was found to be the sole independent predictor of major adverse cardiac events (MACE) [43]. A cut-off value of −14% for GLS showed its potential to predict appropriate ICD therapy in a population of high-risk HCM patients [40], which was further confirmed in larger and more heterogonous HCM patients [1]. GLS of >x15% possessed a significantly superior value over clinical and standard echocardiographic parameters for the hard endpoint of all-cause mortality and appropriate ICD therapy [1]. In another study on HCM patients, GLS of >−15.6% was accompanied by an increased risk of cardiac death, heart failure admission, and appropriate ICD therapy [44].

In general, STE-derived GLS seems to provide valuable information regarding the incidence of arrhythmia and discrimination of high-risk patients. Such a readily available parameter should be highly regarded as a prominent tool for improving risk stratification. Also, GLS improves the identification of low-risk patients which is beneficial in starting medical therapy, following up the outpatients' visits, screening for SCD, and indicating appropriate ICD therapy individuals [1].

This study suffers from some limitations. It was not feasible to compare our echocardiographic findings with data derived from other machines of different vendors as disparities were reported in this area. It should be noted that ICD data may overestimated the reality since self-terminating ventricular arrhythmias were also included. CMR was not performed for all the participants, and accordingly, this makes a lack of comparison of CMR findings with that of STE. The study might be more valued upon the existence of a control group with healthy individuals. Also, a more extended follow- up period and larger sample population increase the strength and generalizability of the present findings, especially regarding determining a sharp cut-off value.

## 5. Conclusion

GLS is a helpful indicator in finding appropriate HCM patients who are candidates of ICD therapy substantiating the strong association of GLS with fatal arrhythmias and sudden cardiac death. Indeed, GLS is especially useful in patients with normal echocardiographic parameters such as EF, LA diameter, left ventricular wall thickness, MWT, and aortic root.

## Figures and Tables

**Table 1:** Medications taken by the patients at the time of enrollment.

Medication class	Number of patients	Percentage (%)
Beta-blockers (excluded)	0	0
Calcium channel blockers (excluded)	0	0
Diuretics (excluded)	0	0
Statins	18	52.90
Aspirin or anticoagulants	12	35.30
ACE inhibitors or ARBs	10	29.40

**Table 2:** STE-derived echocardiographic parameters of the participants.

Echocardiographic parameters	Mean ± SD	Range
LVEDD (mm)	4.8 ± 0.9	2.9–6.9
LVESD (mm)	4.7 ± 1.0	1.4–5.8
IVST (mm)	2.6 ± 0.9	0.8–4.5
PWT (mm)	1.5 ± 0.7	0.7–4.5
MWT (mm)	2.6 ± 0.9	0.8–4.5
LVEF (%)	54.7 ± 11.1	25.0–69.0
LA-D (mm)	3.9 ± 0.8	2.5–5.7
E/A ratio	0.9 ± 0.1	0.5–1.0
GLS (%)	−13.9 ± 5.2	−5.7–−22.2
Aorta root (mm)	3.1 ± 0.4	2.4–3.8

STE: speckle-tracking echocardiography; LVEDD: left ventricular end-diastolic diameter; LVESD: left ventricular end-systolic diameter; IVST: interventricular septal thickness; PWT: posterior wall thickness; MWT: maximum wall thickness; LVEF: left ventricular ejection fraction; LA-D: left atrium diameter; E/A ratio: early to late (A) ventricular filling velocities ratio; GLS: global longitudinal strain. Due to the normality of the right ventricular parameters, data were not reported.

**Table 3:** Comparison of echocardiographic parameters between groups A and B.

	Group A (*N* = 13)	Group B (*N* = 22)	*P* value
LVEDD (mm)	5.0 ± 1.3	4.6 ± 0.4	0.321^*∗∗*^
LVESD (mm)	3.3 ± 1.5	2.3 ± 0.5	0.892^*∗*^
IVST (mm)	2.6 ± 1.2	2.7 ± 0.6	0.792^*∗∗*^
PWT (mm)	1.6 ± 0.9	1.4 ± 0.4	0.254^*∗*^
MWT (mm)	2.6 ± 1.2	2.7 ± 0.6	0.792^*∗∗*^
LVEF (%)	49.5 ± 14.1	57.8 ± 7.6	0.133^*∗*^
LA-D (mm)	4.2 ± 1.0	3.7 ± 0.7	0.138^*∗∗*^
E/A ratio	0.9 ± 0.1	0.9 ± 0.1	0.827^*∗*^
GLS (%)	−8.8 ± 2.8	−17.0 ± 3.8	**<0.001 ** ^*∗*^
Aorta root (mm)	3.2 ± 0.5	3.1 ± 0.4	0.456^*∗*^

LVEDD: left ventricular end-diastolic diameter; LVESD: left ventricular end-systolic diameter; IVST: interventricular septal thickness; PWT: posterior wall thickness; MWT: maximum wall thickness; LVEF: left ventricular ejection fraction; LA-D: left atrium diameter; E/A ratio: early to late (A) ventricular filling velocities ratio; GLS: global longitudinal strain. Data are presented as mean ± SD. ^*∗*^Mann–Whitney *U* test and ^*∗∗*^independent sample t-test. Bold value implies statistical significance.

**Table 4:** The potential of different variables in predicting VT incidence.

	*B*	SE	Wald	df	*P*	Exp (*B*)	95% CI	
							Lower	Upper
Gender	0.121	2.58	0.002	1	0.96	1.12		
GLS (%)	−0.56	0.26	4.58	1	**0.032**	0.57	0.007	176.783
LVEDD (mm)	−0.62	0.97	0.41	1	0.52	0.53	0.345	0.954
PWT (mm)	−0.37	1.57	0.06	1	0.81	0.69	0.081	3.558
MWT (mm)	1.21	1.21	0.99	1	0.32	3.36	0.032	14.918
LVEF (%)	−0.08	0.07	0.97	1	0.32	0.93	0.310	36.447
Constant	−2.265	8.04	0.08	1	0.78	0.1	0795	1.07

VT: ventricular arrhythmia; S.E: standard error; df: degree of freedom; CI: confidence interval; GLS: global longitudinal strain; LVEDD: left ventricular end-diastolic diameter; PWT: posterior wall thickness; MWT: maximum wall thickness; LVEF: left ventricular ejection fraction. Bold value implies statistical significance.

## Data Availability

The data that support the findings of this study are available from the corresponding author upon reasonable request.
